# Serial KL-6 measurements in COVID-19 patients

**DOI:** 10.1007/s11739-020-02614-7

**Published:** 2021-01-16

**Authors:** Miriana d’Alessandro, Laura Bergantini, Paolo Cameli, Giuseppe Curatola, Lorenzo Remediani, David Bennett, Francesco Bianchi, Felice Perillo, Luca Volterrani, Maria Antonietta Mazzei, Elena Bargagli, Siena COVID, Nicola Lanzarone, Francesca Montagnani, Anna Perrone, Federico Franchi, Sabino Scolletta, Serafina Valente, Lucia Migliorini, Barbara Rossetti, Cecilia Vagaggini, Pier Leopoldo Capecchi, Maria Grazia Cusi, Bruno Frediani, Egidio Mastrocinque, Matteo Cameli, Marco Antonio Bellini, Arianna De Lalla, Andrea Melani, Nicola De Stefano, Barbara Porchia

**Affiliations:** 1 Respiratory Diseases and Lung Transplantation, Department of Medical and Surgical Sciences & Neurosciences, Siena University Hospital, Siena, Italy; 2 Diagnostic Imaging Section, Department of Medical and Surgical Sciences & Neurosciences, Siena University Hospital, Siena, Italy; 3 Dipartimento Di Medicina Clinica E Scienze Immunologiche, UOC Malattie Respiratorie, Policlinico Le Scotte, Viale Bracci, 53100 Siena, Italy

**Keywords:** KL-6, Biomarker, COVID-19, Prognosis

## Abstract

SARS-CoV2-induced direct cytopathic effects against type II pneumocytes are suspected to play a role in mediating and perpetuating lung damage. The aim of this study was to evaluate serum KL-6 behavior in COVID-19 patients to investigate its potential role in predicting clinical course. Sixty patients (median age IQR, 65 (52–69), 43 males), hospitalized for COVID-19 at Siena COVID Unit University Hospital, were prospectively enrolled. Twenty-six patients were selected (median age IQR, 63 (55–71), 16 males); all of them underwent follow-up evaluations, including clinical, radiological, functional, and serum KL-6 assessments, after 6 (*t*1) and 9 (*t*2) months from hospital discharge. At *t*0, KL-6 concentrations were significantly higher than those at *t*1 (760 (311–1218) vs. 309 (210–408) p = 0.0208) and *t*2 (760 (311–1218) vs 324 (279–458), *p* = 0.0365). At *t*0, KL-6 concentrations were increased in patients with fibrotic lung alterations than in non-fibrotic group (755 (370–1023) vs. 305 (225–608), *p* = 0.0225). Area under the receiver operating curve (AUROC) analysis showed that basal KL-6 levels showed good accuracy in discriminating patients with fibrotic sequelae radiologically documented (AUC 85%, *p* = 0.0404). KL-6 concentrations in patients with fibrotic involvement were significantly reduced at *t*1 (755 (370–1023) vs. 290 (197–521), *p* = 0.0366) and *t*2 (755 (370–1023) vs. 318 (173–435), *p* = 0.0490). Serum concentrations of KL-6 in hospitalized COVID-19 patients may contribute to identify severe patients requiring mechanical ventilation and to predict those who will develop pulmonary fibrotic sequelae in the follow-up.

**Supplementary Information:**

The online version contains supplementary material available at 10.1007/s11739-020-02614-7.

## Introduction

Krebs von den Lungen-6 (KL-6) is a serum high molecular weight glycoprotein, increased in many interstitial lung diseases (ILDs), including idiopathic pulmonary fibrosis and hypersensitivity pneumonitis [1–3]. It is mainly produced by damaged or regenerating alveolar type II pneumocytes and its serum concentrations are regarded as biomarker of lung epithelial damage (reff). The prognostic value of peripheral KL-6 in ILD has been validated as well as its promising value in predicting the response to antifibrotic therapies [4, 5]. Moreover, KL-6 has been also proposed as bioindicator of acute respiratory distress syndrome (ARDS) and infective pneumonia (ref). Since the outbreak of the severe acute respiratory syndrome coronavirus 2 (SARS-CoV-2) pandemic, KL-6 has also been proposed as a prognostic marker for this disease [6–9]. The pathogenesis of COVID-19 (as the lung disease caused by SARS-CoV-2 is defined) is not entirely clear [10], although it is postulated that elevated serum concentrations of proinflammatory cytokines and oxidative stress mediators contribute to lung injury, facilitating the onset of an acute respiratory syndrome (similar to ARDS) [11]. Host susceptibility and virus-induced direct cytopathic effects against type I and II pneumocytes are suspected to play a crucial role in mediating and perpetuating lung damage [12–14].

Our research group first reported elevated serum concentrations of KL-6 in critical COVID-19 patients and our results were soon confirmed by two other papers [8, 9]. However, no data are currently available on KL-6 role in the follow-up of COVID-19 patients or its potential predictive value for fibrotic lung alteration development.

The aim of this study was to evaluate serum KL-6 behavior in a population of COVID-19 hospitalized patients along the 9-month follow-up of our Centre, to investigate its potential role in predicting clinical course of disease.

## Materials and methods

### Study population

Sixty patients (median IQR, 65 (52–69), 43 males), all hospitalized for COVID-19 at Siena COVID Unit University Hospital, were prospectively enrolled. Fifteen of these patients need intensive care unit (ICU) and mechanical ventilation. Our sixty patients underwent serum sampling specifically for KL-6 assessment on admission (*t*0). Patients who did not give informed consent to the study or who had a previous diagnosis of interstitial lung disease or chronic obstructive pulmonary disease were excluded.

After 6 (*t*1) and 9 (*t*2) months from hospital discharge, twenty-six patients (median age (IQR) 63 (55–71) years, 16 males) underwent follow-up evaluations including physical examination, lung function tests, diffusing capacity of the lung for carbon monoxide (DLCO), blood gas analysis and high-resolution computed tomography (HRCT) of the chest. CT features (fibrotic interstitial lung abnormalities, ground-glass opacities, and air-trapping) were evaluated by on-site radiologists, experienced in interstitial lung diseases. All patients gave their written informed consent to the study for clinical data collection. The study was approved by our local ethics committee (C.E.A.V.S.E Markerlung 17,431).

### KL-6 assay

Serum samples were obtained from all patients on admission, before any biological treatment or intravenous infusion of high-dose steroids or invasive ventilation, and from 26 patients at follow-up, 6 and 9 months after discharge from hospital. Serum concentrations of Krebs von den Lungen-6 were measured by KL-6 reagent assay (Fujirebio Europe, UK), as previously reported [2, 3, 9, 15, 16]. The principle of the assay is agglutination of sialylated carbohydrate antigen with KL-6 mAb by antigen–antibody reaction. The change in absorbance was measured to determine serum concentrations of KL-6, which were expressed in U/ml. A cut-off value of 465 U/ml was applied, as previously reported [17].

### Lung function tests

Lung function tests were performed according to ATS/ERS standards [18] using a Jaeger body plethysmograph with corrections for temperature and barometric pressure. Forced expiratory volume in the first second (FEV1), forced vital capacity (FVC) and diffusing capacity of the lung for carbon monoxide (DLCO) were recorded.

### Statistical analysis

The data did not show a normal distribution. Results were reported as median (IQR), unless otherwise stated. One-way ANOVA nonparametric test (Kruskal–Wallis test) and Dunn’s post test were used for multiple comparisons. The Mann–Whitney test was used to compare pairs of variables. Wilcoxon matched pairs signed rank test was used to compare variables of the same patient at *t*0 and *t*1. The Chi-squared test was used for categorical variables as appropriate. Statistical analysis and graphic representation of the data were performed with GraphPad Prism 8.0 software.

## Results

Table 1 shows the main characteristics of our COVID-19 population at *t*0 (hospital admission). Serum concentrations of KL-6, PFT and blood gas analysis parameters were collected (Table1).

**Table 1:** Main characteristics of COVID19 population including age, gender, smoking habit, BMI, blood gas analysis values, and KL-6 serum concentrations

Parameters	COVID19 patients (*n* = 60)
Age (median *IQR*)	65 (52–69)
Gender, M/F	43/17
Smoking habit (never/former)	38/22
BMI (kg/m^2^)	26 (25–28)
SO2%	97 (96–98)
PaCO2 mmHg	37 (33–39)
PaO2 mmHg	86 (79–92)
FC bpm	74 (71–81)
T0 KL-6 U/ml	760 (311–1218)

At each sampling time, the study population did not differ significantly in terms of sex, age or comorbidities, whereas serum KL-6 concentrations were higher at hospitalization (*t*0) than at *t*1 (760 (311–1218) vs 309 (210–408) *p* = 0.0208) and *t*2 (760 (311–1218) vs 324 (279–458), *p* = 0.0365). About PFT parameters, we did not collect these data at *t*0 in hospitalized patients affected by COVID19.

According to CT radiological features, patients were classified according to the evidence of fibrotic lung alterations (including ground-glass opacities, linear thickening, or organizing pneumonia areas).

**Fig. 1:**
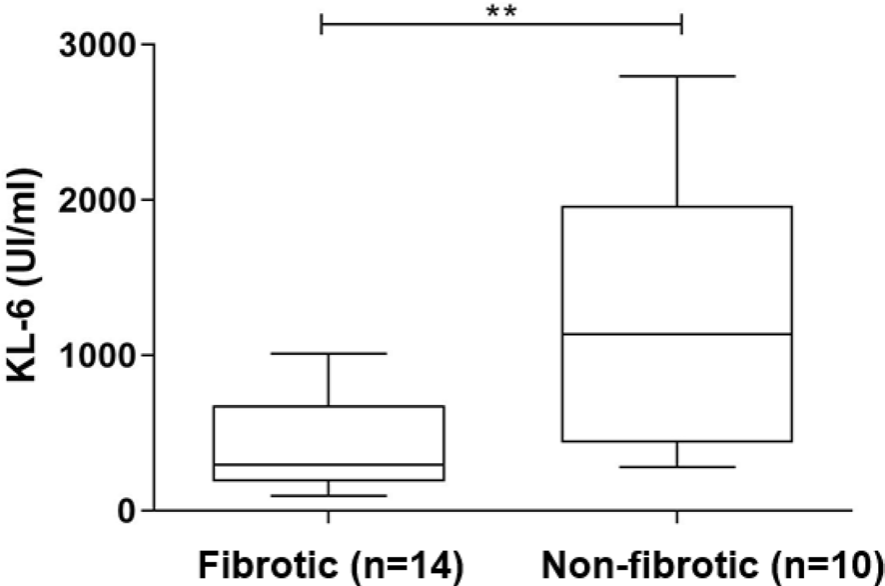
KL-6 concentrations at *t*0 in serum from COVID19 patients divided according to radiological fibrotic interstitial lung abnormalities after 6 months of hospital discharge

**Fig. 2:**
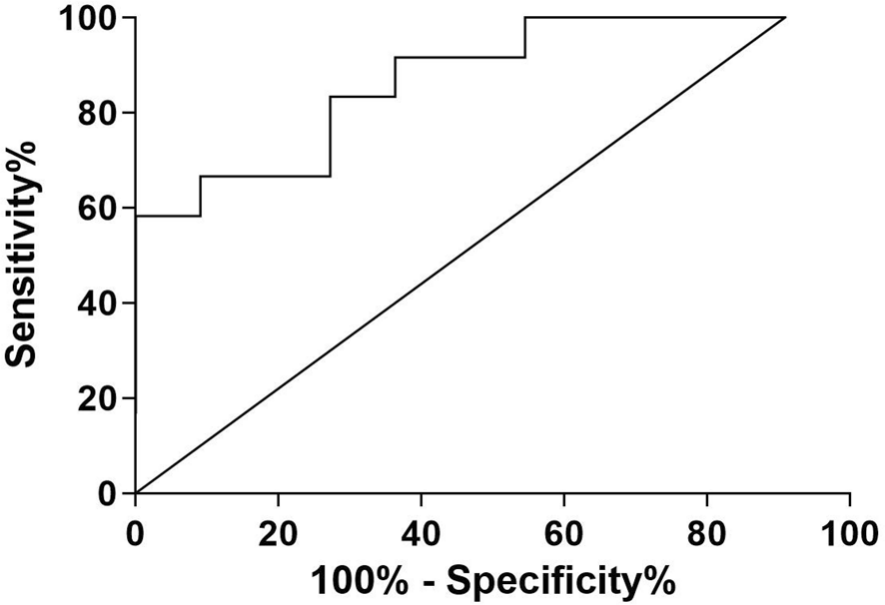
Receiver operating curve analysis showed that, basal KL-6 had good accuracy in discriminating patients with HRCT evidence of fibrotic interstitial lung abnormalities

At *t*0, KL-6 serum concentrations were significantly increased in fibrotic (*n* = 14) than non-fibrotic (*n* = 12) group (755 (370–1023) vs 305 (225–608), *p* = 0.0225) (Fig. 1). Area under the receiver operating curve (AUROC) analysis showed that basal KL-6 had good accuracy in discriminating patients with HRCT evidence of fibrotic interstitial lung abnormalities (AUC = 85%, Std. Error 0.1080, 95%CI 64–100, *p* = 0.0404) (Fig. 2). The best cut-off value for KL-6 concentrations was 455U/ml (75% sensitivity and 80% specificity). At *t*1 and *t*2, no differences of demographic features, blood gas analysis values, and PFT parameters were observed between the two groups (data available). KL-6 concentrations in fibrotic group significantly reduced at *t*1 (755 (370–1023) vs 290 (197–521), *p* = 0.0366) and *t*2 (755 (370–1023) vs 318 (173–435), *p* = 0.0490) (Table 2). Interestingly, three patients maintained very high KL-6 concentrations after 6 and 9 months of follow-up (median IQR, *t*0: 1006 (1000–1011); *t*1: 595 (570–1799); *t*2: 527 (498–1373)), showing simultaneous HRCT evidence of interstitial lung involvement (Fig. 1S). Patients with no fibrotic lung sequelae at HRCT of the chest showed normal KL-6 concentrations at follow-up.

**Table 2:** The main characteristics of our COVID-19 population at follow-up divided according to fibrotic abnormalities, including serum concentrations of KL-6

Parameters	Fibrotic (*n* = 14)	Non-fibrotic (*n* = 12)	*p* value
Age (median *IQR*)	62 (52–71)	64 (49–69)	0.6571
Gender, M/F	12/2	7/5	0.7156
Smoking habit (never/former)	9/5	7/5	0.5541
BMI (kg/m^2^)	25 (24–28)	26 (24–28)	0.4349
Pulmonary function parameters (median, IQR)			
FVC %	89 (82–98)	99 (78–110)	0.1982
FEV1%	91 (85–102)	102 (77–108)	0.2173
DLCO %	80 (78–92)	97 (76–114)	0.0983
T0 KL-6 U/ml	755 (370–1023)^a^	305 (225–608)	0.0225
T1 KL-6 U/ml	290 (197–521)	262 (167–382)	0.2236
T2 KL-6 U/ml	318 (173–435)	320 (214–427)	0.2536

All data were expressed as median IQR. ^a^KL-6 concentrations significantly reduced at *t*1 (*p* = 0.0366) and *t*2 (*p* = 0.0490)

Concerning respiratory function, no significant differences in FVC and/or DLCO percentages were found between patients with and without fibrotic lung alterations at CT scan.

## Discussion

This report first described KL-6 peripheral concentrations in a population of COVID-19 patients after 6 and 9 months from hospitalization discharge together with radiological and functional parameters. Serum concentrations of KL-6 at hospital admission were reported to be significantly increased in severe patients admitted to intensive care unit and requiring intubation with mechanical ventilation, but not in mild-moderate patients with less severe respiratory impairment [9]. This mucin protein has been widely studied in idiopathic pulmonary fibrosis and ARDS patients, but limited data are available on its prognostic potential in infectious viral pneumonia [11–13]. Our interest aroused from the observation that KL-6 was associated with prognosis in ILD and ARDS, reflecting type I and type II alveolar pneumocyte damage. SARS-CoV-2 is known to have specific tropism for alveolar epithelial cells, causing interstitial lung damage, epithelial lung alterations, and regenerative processes, mainly in the acute phase [9]. A significant increase in serum concentrations of KL-6 was demonstrated in patients critically ill with COVID-19 [8, 9] and was further confirmed in our study.

Interestingly, our results demonstrated for the first time a normalization of serum concentrations of KL-6 (< 465U/ml) 6 and 9 months after discharge from hospital. Lung damage due to COVID-19 seemed not to be necessarily progressive, unlike in idiopathic pulmonary fibrosis, where KL-6 tends to increase with IPF progression [1, 4, 5]. Moreover, in the majority of patients with fibrotic sequelae, the decrease and substantial normalization of serum KL-6 concentrations may reflect the amelioration of the clinical status, suggesting that fibrotic interstitial lung abnormalities related to COVID-19 are not associated with persistent active epithelial damage and consequent aberrant fibrogenesis. On the contrary, those patients who developed severe persistent fibrotic lung sequelae at HRCT showed persistent high levels of KL-6 in the follow-up, implicating that this biomarker may be useful in the mid-long-term management of these patients. However, these data will be further evaluated in a longer follow-up to be confirmed. Moreover, in our population, lung volumes were almost preserved at follow-up as well as DLCO percentages, differently from a recent follow-up study reporting functional alterations in more than 25% of patients, albeit after a shorter follow-up of 3 months [19], and this aspect may surely influence the heterogeneity of our population.

Thus, this study contributes to the definition of the natural course of COVID-19 as the normalization of peripheral KL-6 concentrations, recorded 6 and 9 months after the acute phase of SARS-CoV-2 infection, suggesting a non-progressive fibrotic lung involvement in the majority of patients.

Serum concentrations of KL-6 seemed to reflect lung involvement in COVID-19 patients as reflected by HRCT features at 6-month follow-up.

In conclusion, increased serum concentrations of KL-6 in hospitalized COVID-19 patients may help to early discriminate severe patients requiring mechanical ventilation and predict those developing fibrotic lung sequelae in the follow-up.

## Supplementary Information

Below is the link to the electronic supplementary material.

Supplementary file1 (DOC 120 KB)

## Abbreviations

KL-6: Krebs von den Lungen-6
ILDs: Interstitial lung diseases
