# Supplementary material for: Serial KL-6 measurements in COVID-19 patients
Source: Intern Emerg Med. 2021 Jan 16;16(6):1541–5. doi: 10.1007/s11739-020-02614-7 (PMC7811154; doi:10.1007/s11739-020-02614-7)
Supplement: Supplementary file 1 — Supplementary file1 (DOC 120 KB) [file 11739_2020_2614_MOESM1_ESM.doc]

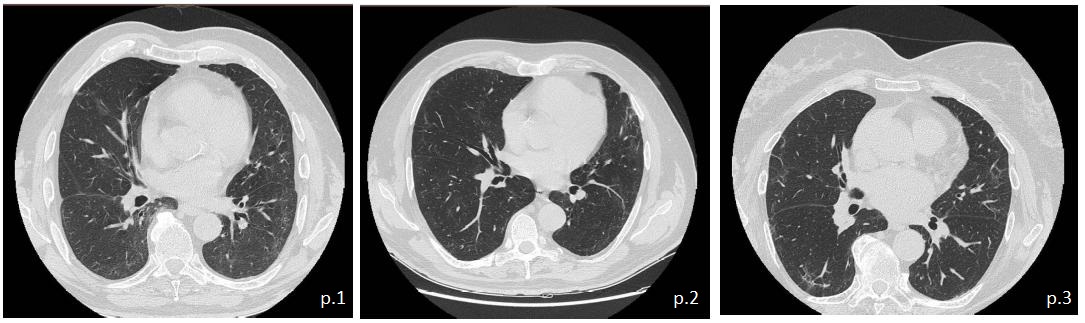
Figure s1. CT scans of patients with fibrotic strends related to COVID19. All CT scans were performed at six months follow-up.
